# Association between lower uterine wall thickness measured at 18–22 weeks of gestation and risk of Preterm Birth: a prospective cohort study

**DOI:** 10.1186/s12884-022-04902-w

**Published:** 2022-08-05

**Authors:** Piengbulan Yapan, Prapat Wanitpongpan, Nawiya Sripang

**Affiliations:** Division of Maternal-Fetal Medicine, Department of Obstetrics and Gynecology, Faculty of Medicine Siriraj Hospital, Mahidol University, 2 Prannok Road, Bangkok Noi, Bangkok, Thailand

**Keywords:** Preterm labour, Lower uterine wall thickness, Prediction, Pregnancy

## Abstract

**Background:**

Preterm labour prediction has been relied on history of previous preterm birth and cervical length of current pregnancy. However, universal cervical length measurement has some limitation. We aim to find a surrogate marker of cervical length to close the gap in preterm prevention program and lower uterine wall thickness seems promising. We generate the nomogram of lower uterine wall thickness during 18–22 weeks of gestation and evaluate the accuracy of LUW thickness as a predictor of preterm delivery before 37 weeks.

**Methods:**

This prospective cohort study included 524 Thai singleton pregnant women at 18–22 weeks of gestation between November 2016 and October 2017. After signing informed consent, transabdominal ultrasonography was performed to examine fetal anatomical structures and to measure LUW thickness. The results were blinded to the caregivers. The outcomes of all pregnancies were followed. The LUW thickness at 10th percentiles was established and was correlated with the outcomes of pregnancy. The performance of LUW thickness at 10th percentile as a predictor of preterm delivery was calculated. The intra-observer and inter-observer reliability of measurement were assessed by intraclass correlation coefficient and Bland-Altman plot.

**Results:**

Of the 524 pregnant women, 64 (12.2%) delivered before 37 weeks of gestation. The reference value of lower uterine wall thickness at 18–22 weeks was established. Mean and 10th percentile of LUW thickness were 6.2 and 4.5 mm respectively. The inter-observer and intra-observer variation of measurement were small (intraclass correlation coefficient = 0.926 and 0.989 respectively). Using LUW thickness at less than 4.5 mm as a predictor of preterm delivery, we found a 2.37 folds increased risk of preterm delivery after adjustment of other factors (*p *= 0.037). Sensitivity, specificity, positive predictive value and negative predictive value were 14% (95% CI: 6.64–25.02), 92.8% (95% CI: 90.06–95.12), 22.5% (95% CI: 12.66–36.76) and 88% (95% CI: 86.92–89.08) respectively.

**Conclusions:**

The measurement of LUW thickness by transabdominal ultrasonography is feasible and reproducible. The risk of delivery before 37 weeks of gestation is increased significantly if the LUW thickness at 18–22 weeks is less than 4.5 mm.

**Trial registration:**

The study protocol was approved by institutional ethical committee (COA No. Si 657/2016).

**Supplementary Information:**

The online version contains supplementary material available at 10.1186/s12884-022-04902-w.

## Background

Preterm birth is one of the major global health problems and is a major cause of neonatal death. Every year, approximately 15 million babies are born prematurely and 1 million of them died [1]. Those who survived suffer many short-term and long-term complications [2]. History of prior preterm birth and short cervical length (CL) at mid-trimester have been proven to be associated with this condition [3–8]. However, transvaginal ultrasonography is still limited in Thailand due to unavailability of the instruments in some hospitals and uncomfortable feelings of the pregnant women towards this procedure. As a result, the screening and preventive strategy are inevitably suboptimal.

The study showed that during the 2nd and 3rd trimester of pregnancy lower uterine wall (LUW) thickness is maintained by some mechanisms and then becomes thinner after 28–32 weeks of gestation [9, 10]. When parturition occurs, frequent uterine contractions will lead to the change in the cervix and the LUW is progressively thinning [11–13]. In contrast, preterm labor starts with ripening of the cervix and then frequent uterine contractions follow. Lower uterine wall lies closely to the cervix and we hypothesized that the changes that originated in the cervix could involve the adjacent LUW as well. This idea was confirmed by the study of Woraboot et al. which demonstrated a highly correlation between LUW thickness and CL at mid-trimester [14]. Sfakianaki et al. also reported that thinning of the LUW occurs earlier in twin pregnancies destined to deliver prematurely [15].

The authors expected that LUW thickness could be a surrogate marker to predict preterm delivery when CL is not available. This study aimed to generate the reference value of LUW thickness at 18–22 weeks of gestation in Thai singleton pregnant women and to assess the association between LUW thickness and risk of preterm delivery before 37 weeks.

## Methods

We conducted a prospective cohort study between November 2016 and October 2017. The study protocol was approved by institutional ethical committee (COA No. Si 657/2016). Thai singleton pregnant women at 18–22 weeks of gestation who were scheduled for routine 2nd trimester fetal anatomical scan at Maternal-Fetal Medicine unit, Siriraj Hospital were invited to participate in this study. The exclusion criteria were age < 18 years old, previous uterine or cervical surgery, the presence of myoma uteri, previous preterm birth, fetal anomalies, indicated preterm birth of the current pregnancy, receiving progesterone therapy, and outcome data and/or LUW thickness was not obtainable. The gestational age was defined by correct menstrual history or crown-lump length measurement during the 1st trimester. Due to the lacking of standard measurement of LUW thickness, the investigators improvised the method from previous studies [16, 17]

**Fig. 1:**
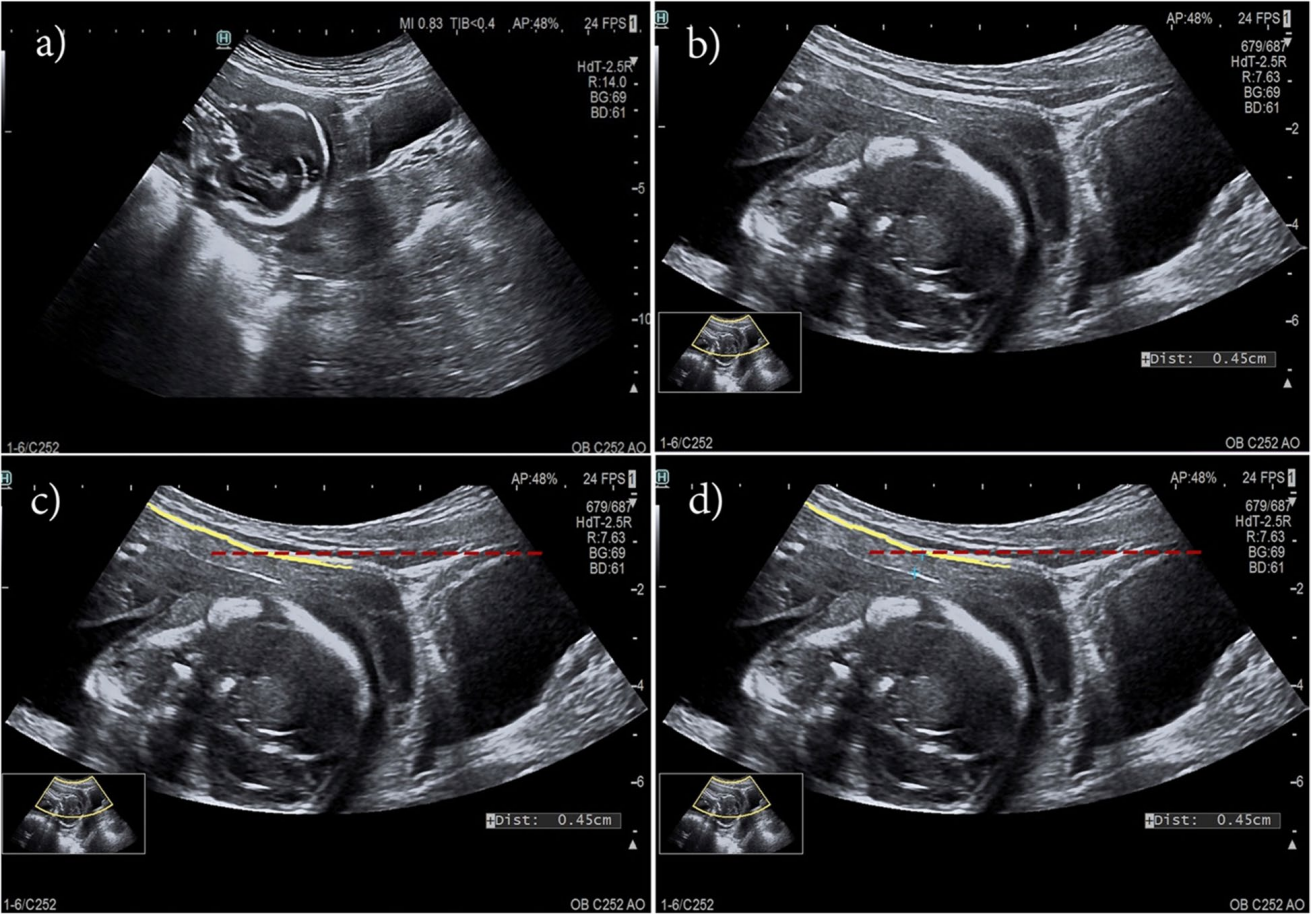
The measurement of the LUW thickness. **a** An ultrasound transducer was placed in sagittal plane just above the pubic symphysis to visualize the urinary bladder, cervix with visible cervical canal, and LUW. **b** The picture was enlarged until LUW and the urinary bladder occupied the majority of the screen. **c** A virtual red dashed line was drawn in horizontal plane between the dome of urinary bladder and LUW to mark a point for measurement. The yellow line represented the peritoneum that covered the LUW. **d **The calipers were placed in inner-to-inner fashion to measure the LUW thickness

 After giving their written informed consents, the participants were advised to urinate and lied on supine position. The LUW thickness was measured by transabdominal ultrasonography by the first investigator (P.Y). The LUW thickness measurement techniques were described in Fig. 1 and as follow : the 5 MHz 2D ultrasound probe attached to Voluzon E8 ultrasound platform (General Electric, Zipf, Austria) was placed in sagittal plane just above the pubic symphysis. After a little adjustment, the picture of the urinary bladder with little residual urine and the cervical canal in mid-sagittal plane would be clearly visualized. The picture was enlarged until LUW and the urinary bladder occupied the majority of the screen. The virtual horizontal line that connected the dome of the urinary bladder and the LUW was made. LUW thickness was measured at this point perpendicular to the outer surface of the uterine wall. The first caliper was placed just beneath the peritoneum and the other at uterine-amniotic fluid interface (inner-to-inner fashion).

Caution was exercised not to include the surrounding tissue during the measurement. The measurement was performed 3 times and the lowest value was selected for statistical analysis. The measurement was recorded in case record form and was blinded from the caregivers. The detailed fetal anatomical scan was then performed. The characteristics of the participants and outcomes of the pregnancies were recorded for further statistical analysis. The inter-observer and intra-observer variation of measurement were assessed in 40 randomly-selected subjects.

The data was analyzed by PASW statistics 18.0 (SPSS Inc., Chicago, IL, USA). Descriptive statistic was used to describe characteristic data of the participants. Continuous variables were expressed as mean and standard deviation (SD) or median and interquartile range. Categorical data was expressed as number and percentage. The Receiver Operating Characteristics (ROC) curve and Area Under the Curve (AUC) were established for the prediction of preterm and no-preterm events. Univariate and multivariate analysis (logistic regression analysis) were used to assess the association of factors and outcome of interest (preterm delivery). Intraclass correlation coefficient and Bland-Altman plot were used to demonstrate inter-observer and intra-observer reliability of the measurement (presented in Additional file 1). All tests of significance were two-tailed, *p* value < 0.05 was considered statistically significant.

## Results

**Fig. 2:**
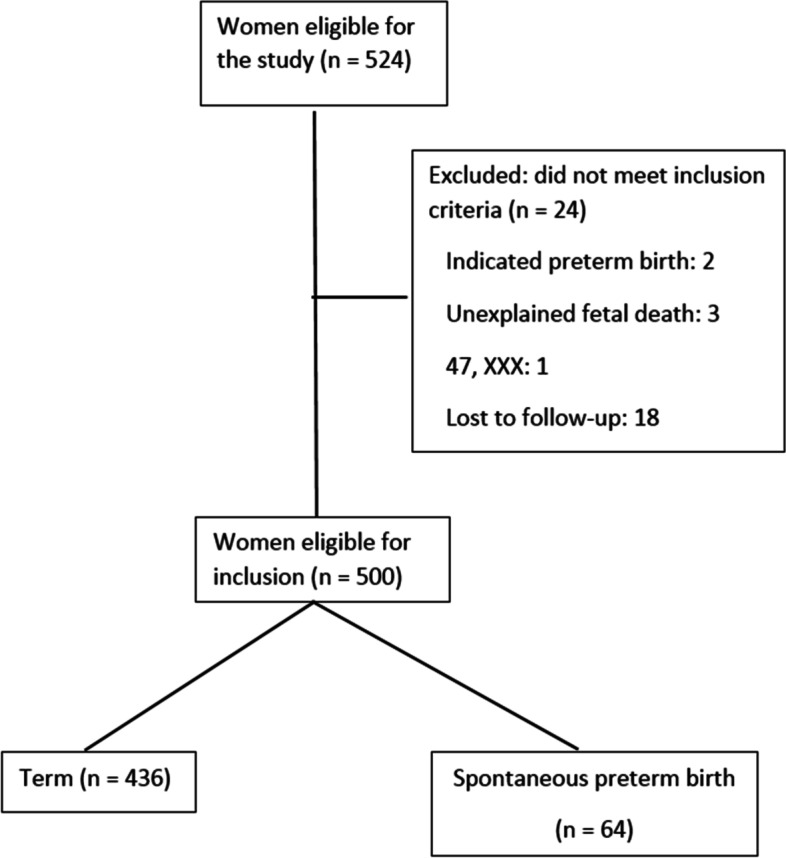
Flowchart summarizing the process of the study

Five hundred and twenty-four pregnant women were recruited for the study. Twenty-four cases were excluded and 500 participants were eligible for analysis (Fig. 2).

Preterm delivery occurred in 64 cases (12.8%). LUW thickness could be obtained from all participants. The baseline characteristics of all participants were shown in Table 1.

**Table 1:** Characteristics of the participants (*n* = 500)

	Term Delivery (*n* = 436)		Preterm Delivery (*n* = 64)		*p*-value
**Characteristics**	**n (%)**	**Mean ± SD or Median (IQR)**	**n (%)**	**Mean ± SD or Median (IQR)**	
**Age (years)**		29.5 ± 6.5		30.3 ± 6.4	0.413^a^
18-24.9	117 (26.8)	21.3 ± 2.0	15 (23.4)	21.5 ± 2.1	0.646^a^
25-34.9	199 (45.6)	29.5 ± 2.9	28 (43.8)	29.6 ± 2.9	0.832^a^
≥ 35	120 (27.5)	37.6 ± 2.3	21 (32.8)	37.3 ± 6.3	0.538^a^
**Parity**					
0	252 (57.8)	0	33 (51.6)	0	0.348^b^
≥ 1	184 (42.2)	1 (1,2)	31 (48.4)	1 (1,2)	0.210^b^
**BMI (kg/m**^2^**)**		22.1 ± 4.0		22.9 ± 4.9	0.183^a^
≤ 18	50 (11.5)	16.8 ± 0.8	5 (7.8)	16.1 ± 1.0	0.091^a^
18–25	300 (68.8)	21.2 ± 1.8	41 (64.1)	20.8 ± 1.9	0.246^a^
> 25	86 (19.7)	28.5 ± 3.3	18 (28.1)	29.4 ± 3.4	0.289^a^
**Current weight (kg)**		58.9 ± 10.7		61.0 ± 13.5	0.156^a^
≤ 60	268 (61.4)	52.3 ± 4.8	35 (54.7)	51.4 ± 5.4	0.292^a^
60.1–80	146 (33.4)	66.4 ± 4.8	20 (31.3)	65.8 ± 3.9	0.540^a^
> 80	22 (5.0)	88.7 ± 7.4	9 (14.1)	87.9 ± 4.1	0.743^a^
**Placental location**					
Anterior	186 (42.7)	-	32 (50.0)		0.283^b^
Posterior	237 (54.4)	-	30 (45.9)		0.285^b^
Fundus	13 (3.0)	-	2 (3.1)		> 0.99^b^
**LUW thickness (mm)**		6.25 ± 1.46		6.02 ± 1.37	0.230^a^
< 4.5	31 (7.1)	3.9 ± 0.4	9 (14.1)	3.9 ± 0.3	0.785^a^
≥ 4.5	405 (92.9)	6.4 ± 1.3	55 (85.9)	6.3 ± 1.1	0.687^a^
**Route of delivery**					
Spontaneous vaginal delivery	263 (60.3)	-	38 (59.4)	-	0.892^b^
Cesarean section	164 (37.6)	-	25 (39.1)	-	0.890^b^
Vacuum extraction	9 (2.1)	-	0	-	-
Forceps extraction	0	-	1 (1.6)	-	-
**Birth weight (g)**		3134.4 ± 429.2		2417.2 ± 480.2	< 0.005^a^
< 1,500	0	-	3 (4.7)	1173.3 ± 324.7	-
1,500–2,499	18 (10.8)	2368.3 ± 145.6	36 (56.3)	2224.0 ± 229.2	0.019^a^
2,500–3,999	402 (85.4)	3119.1 ± 320.4	25 (39.1)	2844.7 ± 283.2	< 0.005^a^
≥ 4,000	16 (3.2)	4380.0 ± 456.2	0	-	-

*P* value < 0.05 is considered statistically significant

^a^t-test

^b^Chi-square test

The value of the LUW thickness at 18–22 weeks of gestation in Thai singleton pregnant women was shown in Table 2. The mean and 10th percentiles of LUW thickness was 6.2 and 4.5 mm, respectively.

**Table 2:** The value of the lower uterine wall thickness measured at 18–22 weeks in Thai singleton pregnant women

	Mean ± SD	Median (IQR)	Range	Percentile			
				**5th**	**10th**	**50th**	**95th**
**Lower uterine wall thickness(mm)** (*N* = 500)	6.2 ± 1.45	6.0 (5.2, 7.1)	3.1–12.0	4.10	4.50	6.00	8.80

**Fig. 3:**
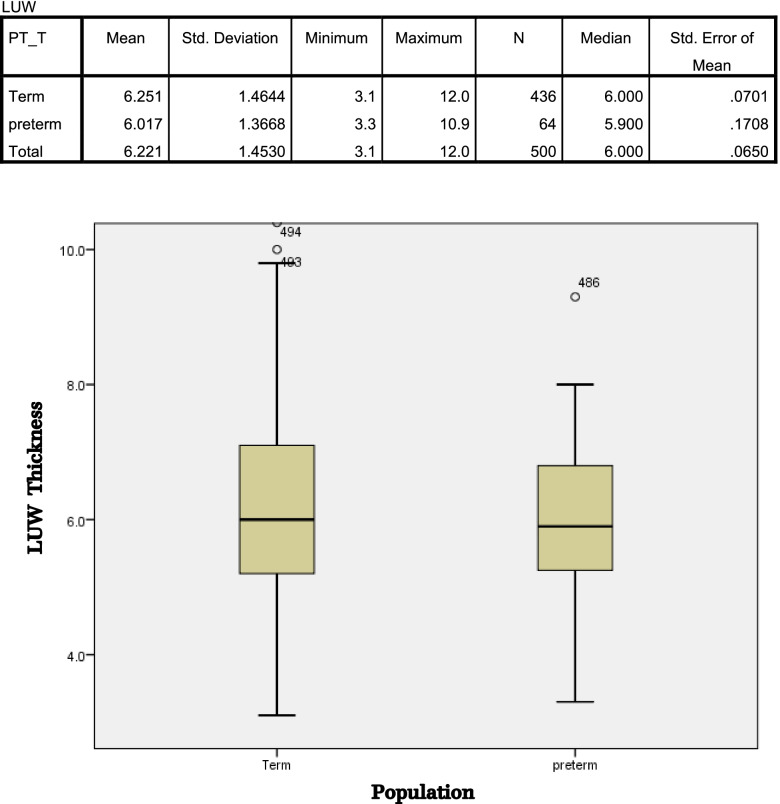
The interquartile range of LUW thickness between the two groups

The interquartile range of LUW thickness of pregnant women who delivered before 37 weeks was established and compared with those who delivered at term. The median of LUW thickness looked similar in both groups, but the distribution of thicker LUW thickness was clearly visible in term delivery group, while the proportion of thinner LUW thickness was more prominent in preterm delivery group (Fig. 3).

**Fig. 4:**
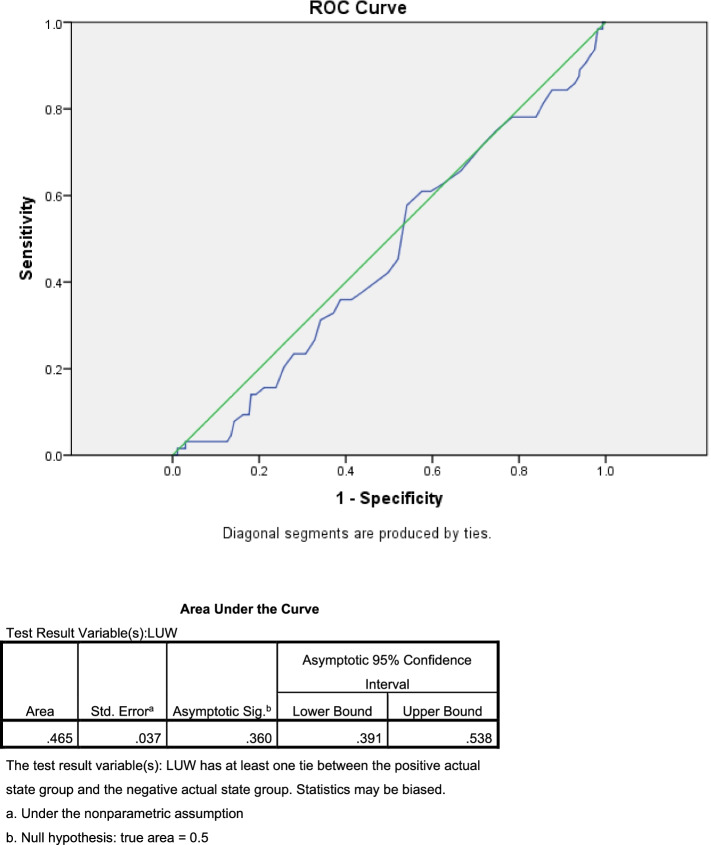
The ROC curve and AUC of LUW thickness

The ROC curve was constructed and its AUC was obtained as shown in Fig. 4.

**Fig. 5:**
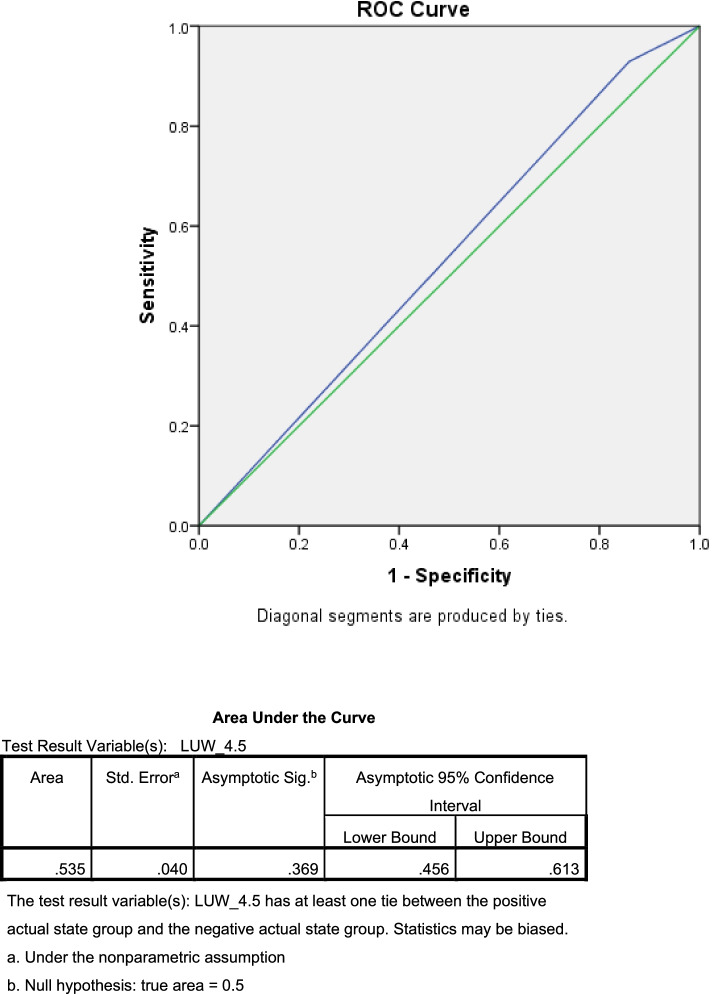
The ROC curve and AUC of LUW thickness in predicting no-preterm events

Owing to the poor performance of LUW thickness as a predictor of PTB demonstrated by ROC curve and AUC, the authors tried to construct the other ROC curve using LUW thickness to predict no-preterm events as shown in Fig. 5.

Once again, the ROC curve did not show any reliable cut-off value and the AUC was 0.53 which implied the poor performance of the marker to predict no-preterm events. Finally, the authors turned to the 10th percentile value of LUW thickness. The LUW thickness at less than 4.5 mm (10th percentile) was further analyzed for its performance as a predictor of predicting delivery before 37 weeks of gestation. The factors that were possibly associated with preterm delivery were maternal age, parity, body mass index (BMI) and LUW thickness less than 4.5 mm as shown in Table 3. After logistic regression analysis, only the LUW thickness less than 4.5 mm significantly increased risk of preterm birth compared with the women whose LUW thickness were greater than or equal to 4.5 mm (OR = 2.37 (95% CI 1.05–5.32), *p* = 0.037). Sensitivity, specificity, positive predictive value (PPV) and negative predictive value (NPV) were 14% (95% CI: 6.64–25.02), 92.8% (95% CI: 90.06–95.12), 22.5% (95% CI: 12.66–36.76) and 88% (95% CI: 86.92–89.08) respectively.

**Table 3:** Factors associated with preterm delivery (*N* = 500)

Factors	Mean ± SD or n (%)		Univariate analysis		Multivariate analysis	
	**Term** (*n* = 436)	**Preterm** (*n* = 64)	***p*-value**	**Crude OR** (95% CI)	***p*-value**	**Adjust OR** (95% CI)
**Age (years)**						
18–24.9	117 (88.6)	15 (11.4)		1		
25–34.9	199 (87.7)	28 (12.3)	0.785	1.10 (0.56, 2.14)		
≥ 35	120 (85.1)	21 (14.9)	0.390	1.37 (0.67, 2.78)		
**Parity**						
0	252 (88.4)	33 (11.6)		1		1
≥ 1	184 (85.6)	31 (14.4)	0.348	1.29 (0.76, 2.18)	0.359	1.29 (0.75, 2.22)
**BMI (kg/m**^2^**)**						
≤ 18	50 (90.9)	5 (9.1)		1		1
18–25	300 (88.0)	41 (12.0)	0.530	1.37 (0.52, 3.63)	0.627	1.28 (0.48, 3.42)
> 25	86 (82.7)	18 (17.3)	0.168	2.09 (0.73, 5.98)	0.205	2.00 (0.69, 5.81)
**LUW thickness**						
≥ 4.5 mm	405 (88.0)	55 (12.0)		1		1
< 4.5 mm	31 (77.5)	9 (22.5)	0.061	2.14 (0.97, 4.73)	0.037	2.37 (1.05, 5.32)

Among 64 prematurely-born babies, 8 cases were diagnosed with respiratory distress syndrome and were admitted to neonatal intensive care unit, 2 cases had sepsis, one case had intracranial hemorrhage. All of these babies survived and were discharged home eventually. The lowest birth weight was 800 g (26 weeks of gestation).

## Discussion

This study aimed to find a surrogate marker of cervical length (CL) to predict the risk of preterm delivery. Our previous study showed a high correlation between LUW thickness and CL at mid-trimester and was the foundation that inspired our team to study more about this parameter. From our experience, LUW thickness is easy to measure, highly reproducible and could be achieved by transabdominal ultrasound machine which is readily available in almost all obstetric settings. We could not advocate that our measurement technique is of exemplary approach and we would be very happy to see the improvement of the measurement in the future. However, at the present time when the consensus and the standard of the measurement of the LUW thickness do not exist, we believe that our technique is easy to follow and not affected or very little affected, if any, by the body contour of the mothers or placental locations. The mean and 10th percentile of the LUW thickness from this study was 6.2 mm (range 3.1-12 mm.) and 4.5 mm respectively which were a little higher than our previous study (mean = 5.4 mm. and 10th percentile value = 4.2 mm.) [14]. We believe that the difference is not a result of different population as the characteristics of the participants in both studies were the same. The difference could possibly be a result of the different number of the subjects in each study. As this study recruited 10 times more subjects than the previous study (500 cases vs. 41 cases), we believe that the values in this study are more reliable.

The ROC curve did not demonstrate any reliable cut-off point and AUC showed that LUW thickness performed poorly as a predictor of PTB. As a result, LUW thickness seems to be ineffective as a surrogate marker for CL for predictive purposes. Further analysis using LUW thickness at 10 percentile (4.5 mm) as a cut-off value, we found that the group with LUW thickness less than this cut-off had 2.37 folds higher risk of preterm delivery before 37 weeks of gestation. Sensitivity, specificity, positive predictive value (PPV) and negative predictive value (NPV) were 14, 92.8, 22.5 and 88% respectively. Again, with quite low sensitivity, LUW thickness did not appear to be a reliable predictor which generally requires high sensitivity to perform well during screening. However, with its high specificity (92.8%) and high NPV (88%) in predicting PTB before 37 weeks, as well as the finding from previous study that it highly correlates with CL, the authors believe that LUW thickness still has some benefit in PTB screening program. Instead of replacing CL, LUW thickness could be used to supplement CL in general pregnant population where transvaginal ultrasonography is not available or the pregnant women deny transvaginal CL measurement. Transvaginal ultrasonography can be selectively performed in those whose LUW thickness is less than 4.5 mm to confirm the CL and actual risk of preterm delivery. We believe that incorporating LUW thickness measurement into routine antenatal care practice could leverage the performance of PTB screening and prevention program.

In this study we tried to study the use of LUW thickness to predict the preterm birth before 34 weeks which is more problematic than late preterm birth. Unfortunately, we did not have enough power to draw any conclusion. From the results of our previous study, it showed that the LUW changed in thickness in the same direction with the length of the cervix. However, that study did not enlighten on the mechanism of that finding. Even so, we thought it’s possible that the LUW which lies next to the cervix becomes thinner as a result of the inflammations originated in the cervix as a starting point of preterm cascade. The remodeling of elastic tissue and reorganization of the extracellular matrix and fluid that resulted in shortening of the cervix before the onset of preterm birth might play the same role at the LUW. Further studies about pathophysiology of preterm labor at molecular level might shed some light on this issue in the future.

Other factors such as older age, parity and high BMI were not significantly associated with preterm birth similar to previous studies [18–21].

The strength of the present study was the large prospective cohort study in singleton pregnancies using LUW thickness measured by transabdominal sonography to predict preterm delivery. The limitation of this study is the lack of conclusion about prediction of early preterm delivery. Further studies in selective high-risk populations e.g. twins pregnancies should be encouraged.

## Conclusions

The measurement of lower uterine wall thickness by transabdominal ultrasonography is feasible and reproducible. Although lower uterine wall thickness less than 4.5 mm as a predictor of PTB before 37 weeks has a low predictive value, it should be considered to supplement PTB screening when transvaginal ultrasonography is not available .

## Supplementary Information

**Additional file 1:** The reliability of LUW thickness measurement.  **Figure 1.** Bland-Altman plot showed the difference of LUWthickness measurement between two operators (Inter-observer reliability)(*N*=40)]. **Figure 2.** Bland-Altman plot showed the differenceof LUW thickness measurement between two measurements of the same operator(Intra-observer reliability) (*N*=46)].

## Data Availability

The datasets used and/or analyzed during the current study available from the corresponding author on reasonable request. To enable a quick and smooth reviewing process, the authors have uploaded those data and materials to google drive. The link for downloading the data and materials is provided below. https://drive.google.com/file/d/16LJAV0pRSZwcExKqOAxy39nBGr8a2TbS/view?usp=sharing.

## References

[1] Preterm birth: World Health Organization; 2015 [updated Nov 2015]. Available from: http://www.who.int/mediacentre/factsheets/fs363/en/.

[2] Patel RM (2016). Short- and Long-Term Outcomes for Extremely Preterm Infants. Am J Perinatol..

[3] Haram K, Mortensen JHS, Wollen AL (2003). Preterm delivery: an overview. Acta Obstet Gynecol Scand.

[4] Kenneth Lim M, Vancouver BC (2011). Ultrasonographic cervical length assessment in predicting preterm birth in singleton pregnancies. J Obstet Gynaecol Can.

[5] American College of Obstetricians and Gynecologists (2012). Prediction and prevention of preterm birth. Practice Bulletin no.130. Obstet Gynecol.

[6] Di Renzo Gian Carlo (2015). FIGO Committee Report. Best practice in maternal–fetal medicine. Int J Gynecol Obstet.

[7] Wanitpongpan P, Sutchritpongsa P, Rongluen S (2015). Cervical length at mid-trimester in Thai women with normal singleton pregnancies. Siriraj Med J.

[8] Society for Maternal-Fetal Medicine Publications Committee, with assistance of Vincenzo Berghella. Progesterone and preterm birth prevention: translating clinical trials data into clinical practice. Am J Obstet Gynecol. 2012;206(5):376–86. 10.1016/j.ajog.2012.03.010 22542113

[9] Durnwald C, Mercer B (2008). Myometrial thickness according to uterine site, gestational age and prior Cesarean delivery. J Matern Fetal Neonatal Med.

[10] Norwitz ER, Mahendroo M, Lye SJ, Resnik R, Lockwood CJ, Moore TR, Greene MF, Copel JA, Silver RM (2019). Physiology of Parturition. Creasy and Resnik’s Maternal-Fetal Medicine: Principle and Practice.

[11] Physiology of labor. In: Cunningham FG, Leveno KJ, Bloom SL, Dashe JS, Hoffman BL, Casey BM, Spong CY, editors. William Obstetrics. 25th ed. New York, McGraw-Hill Education; 2018. p. 400–420.

[12] Kearns PJ (1942). The lower uterine segment: anatomical changes during pregnancy and labour. Can Med Assoc J.

[13] Morrison J (1972). The development of the lower uterine segment. Aust N Z J Obstet Gynaecol.

[14] Woraboot W, Wanitpongpan P, Phaophan A (2019). Correlation of lower uterine segment thickness by transabdominal ultrasonography with cervical length by transvaginal ultrasonography in Thai pregnant women. J Chin Med Assoc.

[15] Sfakianaki AK, Buhimschi IA, Pettker CM, Magloire LK, Turan OM, Hamar BD (2008). Ultrasonographic evaluation of myometrial thickness in twin pregnancies. Am J Obstet Gynecol.

[16] Sambaziotis H, Conway C, Figueroa R, Elimian A, Garry D (2004). Second-trimester sonographic comparison of the lower uterine segment in pregnant women with and without a previous Cesarean delivery. J Ultrasound Med.

[17] Buhimschi CS, Buhimschi IA, Norwitz ER, Sfakianaki AK, Hamar B, Copel JA (2005). Sonographic myometrial thickness predicts the latency interval of women with preterm premature rupture of the membranes and olighydramnios. Am J Obstet Gynecol.

[18] Riley KL, Carmichael SL, Mayo JA, Shachar BZ, Girsen AI, Wallenstein MB (2016). Body mass index change between pregnancies and risk of spontaneous preterm birth. Am J Perinatol.

[19] Astolfi P, Zonta LA (1999). Risks of preterm delivery and association with maternal age, birth order, and fetal gender. Hum Reprod.

[20] Fuchs F, Monet B, Ducruet T, Chaillet N, Audibert F (2018). Effect of maternal age on the risk of preterm birth: A large cohort study. PLoS One.

[21] Schempf AH, Branum AM, Lukacs SL, Schoendorf KC (2007). Maternal age and parity-associated risks of preterm birth: differences by race/ethnicity. Paediatr Perinat Epidemiol.

