# Supplementary material for: Association between lower uterine wall thickness measured at 18–22 weeks of gestation and risk of Preterm Birth: a prospective cohort study
Source: BMC Pregnancy Childbirth. 2022 Aug 5;22:618. doi: 10.1186/s12884-022-04902-w (PMC9354291; doi:10.1186/s12884-022-04902-w)
Supplement: Supplementary file 1 — Additional file 1: The reliability of LUW thickness measurement. Figure 1. Bland-Altman plot showed the difference of LUWthickness measurement between two operators (Inter-observer reliability)(N=40)]. Figure 2. Bland-Altman plot showed the differenceof LUW thickness measurement between two measurements of the same operator(Intra-observer reliability) (N=46)]. [file 12884_2022_4902_MOESM1_ESM.docx]

**The reliability of LUW thickness measurement**

The inter-observer and intra-observer reliability of LUW thickness measurement were high as demonstrated by intraclass correlation coefficient (ICC) of 0.926 and 0.989, respectively.

Bland-Altman plot showed small and non-significant difference between the two operators (0.195 mm., 95%CI -0.44,0.434; p=0.138) (Figure 1) and between the two measurements of the same operator (0.080 mm., 95% CI -0.185, 0.024; p = 0.129) (Figure 2).


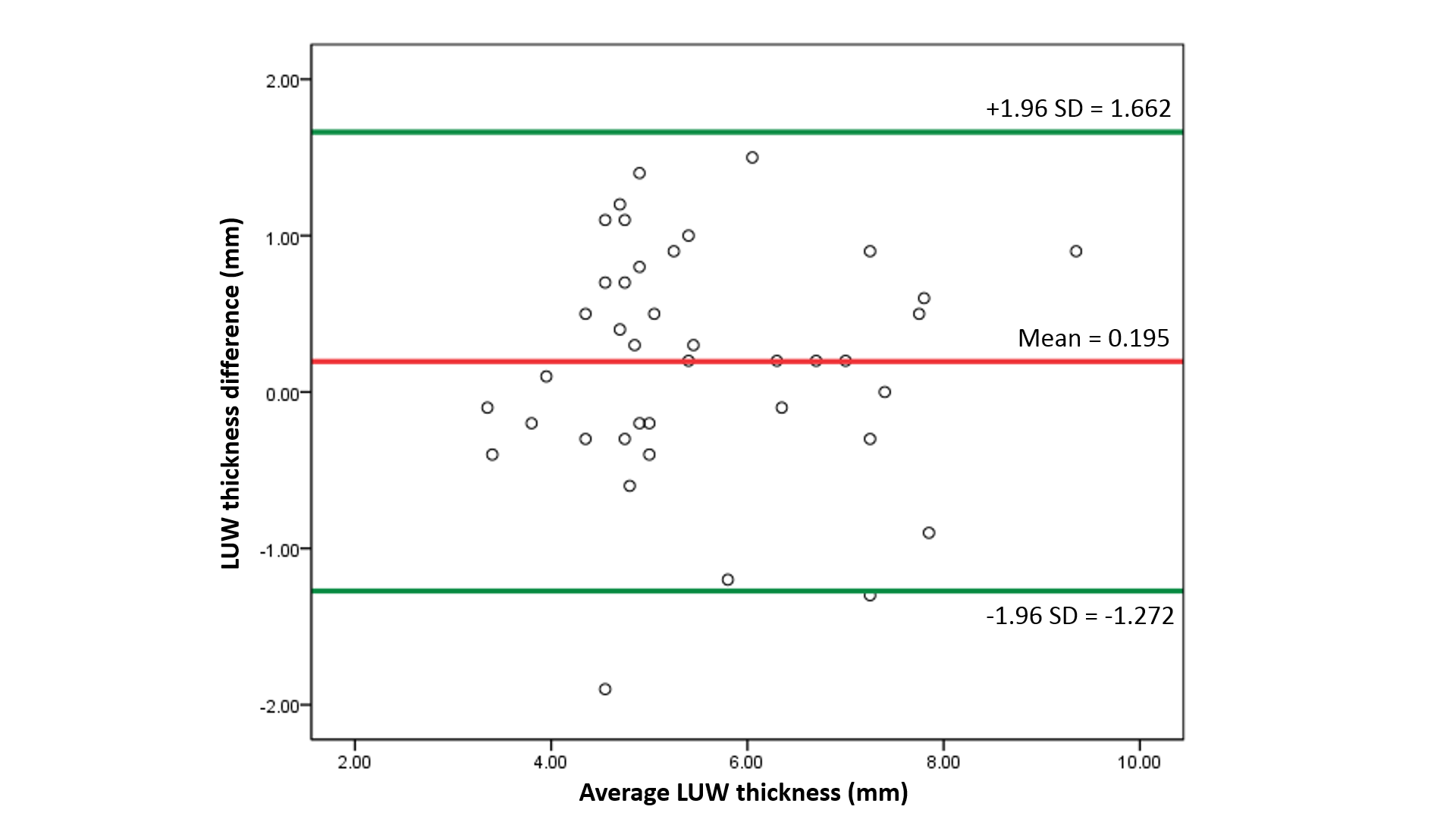


[ Figure 1 : Bland-Altman plot showed the difference of LUW thickness measurement between two operators (Inter-observer reliability) (N=40).]


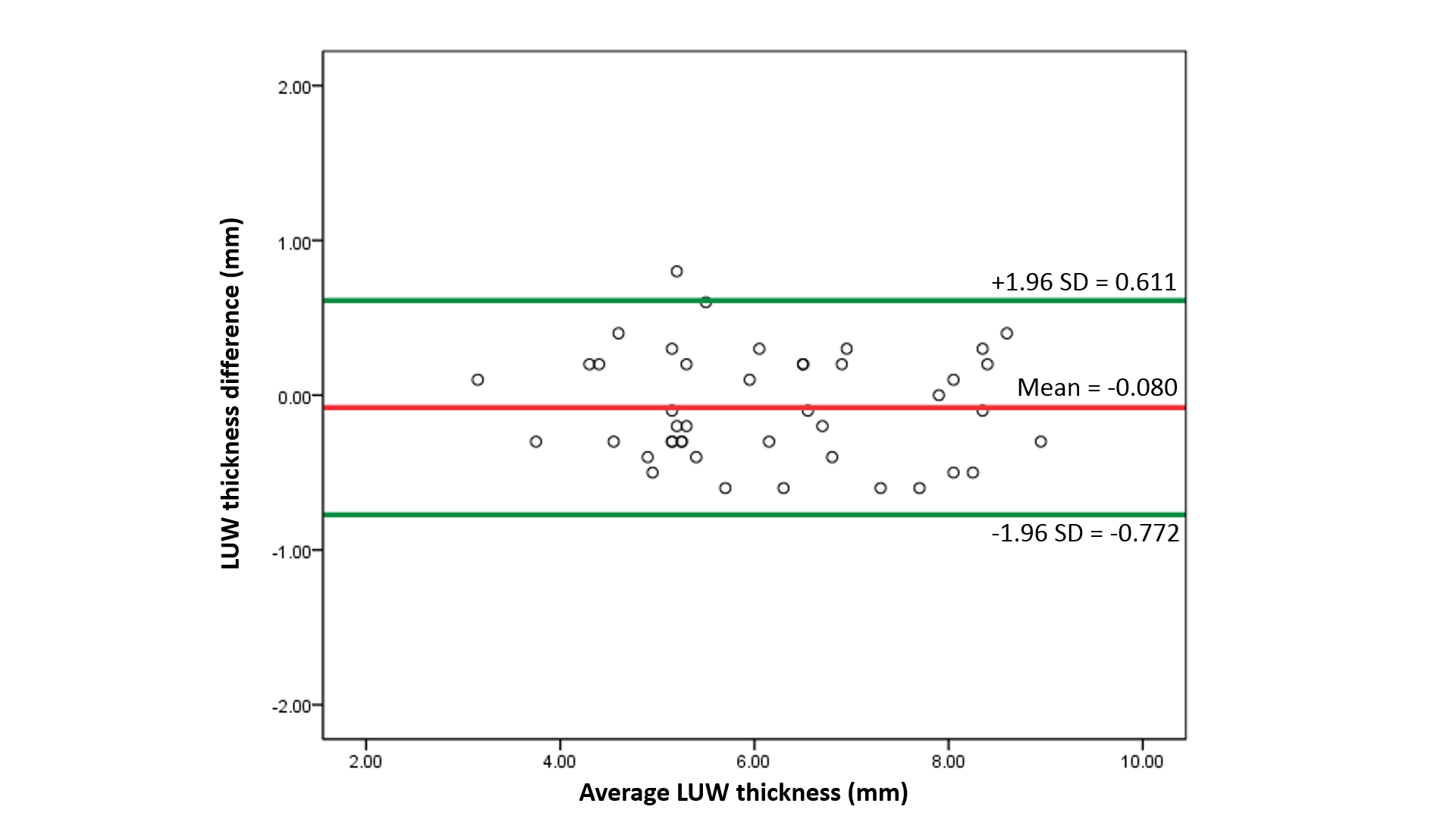


[ Figure 2 : Bland-Altman plot showed the difference of LUW thickness measurement between two measurements of the same operator (Intra-observer reliability) (N=46).]
